# Supplementary material for: Patterns of Intron Gain and Loss in Fungi
Source: PLoS Biol. 2004 Nov 30;2(12):e422. doi: 10.1371/journal.pbio.0020422 (PMC532390; doi:10.1371/journal.pbio.0020422)
Supplement: Table S1 — Also available at http://genes.mit.edu/NielsenEtAl/. (4.3 MB ZIP). [file pbio.0020422.st001.zip › NielsenEtAl/html/95.html]

AN8680.1.NCU00578.1.MG02997.1.FG08777.1


```
 CLUSTAL W (1.82) Multiple Sequence Alignments - Introns Inserted


Sequence 1: NCU00578.1	153 aa
Sequence 2: FG08777.1	162 aa
Sequence 3: MG02997.1	159 aa
Sequence 4: AN8680.1	162 aa
Alignment Length: 163 aa
Number Identitical Residues: 102 aa
Alignment Score (without introns) 4642


MG02997.1 	MATNVSIETSM0GTIVVELYESHAPK~TCKNFSTLASRGYYDGTVFHRVISDFMIQGGDP
NCU00578.1	----------M0GTFTLELYTNHAPK~TCKNFATLADRGYYDSTVFHRIIKDFMIQGGDP
FG08777.1 	MTTNIVLETTM0GSLILELYTTHAPK~TCNNFTTLVRRGYYDNTIFHRIIPNFMVQGGDP
AN8680.1  	MATDVTFDTSM0GSFTVELYNSHAPK0TCKNFATLAQRGYYNNVIFHRIIPNFMVQTGDP
          	 ::.   .::* *:: :*** .**** **:**:**. ****:..:***:* :**:* ***

MG02997.1 	TGTGRGGSSIYGDKFEDEIRQDLKHTGAGILSMANAGPNTNGSQFFITLAPTPWLDGKHT
NCU00578.1	TGTGRGGSSIYGEKFEDEIHPGLKHTGAGVLSMANAGPNTNGSQFFITLAPTPWLDGKHT
FG08777.1 	TGTGRGGSSIFGEKFEDEIDPGLKHTGAGILSMANAGPNTNGSQFFVTLAPTPWLDGKHT
AN8680.1  	TGTGRGGSSIYGEKFEDEIRSDLKHTGAGILSMANSGPNTNGSQFFITLAPTPWLDGKHT
          	**********:*:******  .*******:*****:**********:*************

MG02997.1 	IFGRVSGGMGVVERMGRVQTDKSDRPVTELKIVRANVVEQQ----
NCU00578.1	IFGRVKKGMGVIRRMGMVPTDKEDRPATEVKIVKARVVREEDMEA
FG08777.1 	IFGRVKSGMGTIKRMGLVKTGSEDRPVEEVKIVKARVVEEEEQI-
AN8680.1  	IFGRVKSGMRIIQRMGLVKTNNEDRPLDEVKILRAKVVEAGSEE-
          	*****. **  :.*** * *...***  *:**::*.**.  .
```
